# Supplementary material for: Content-rich biological network constructed by mining PubMed abstracts
Source: BMC Bioinformatics. 2004 Oct 8;5:147. doi: 10.1186/1471-2105-5-147 (PMC528731; doi:10.1186/1471-2105-5-147)
Supplement: Additional File 2 — The original results of the above study (non-essential files are deleted to keep the file size under the limit set by BMC bioinformatics). [file 1471-2105-5-147-S2.bz2 › chilibotAdditionalFile2/dip05/728ID9822594E18078/html/TOLC_HLYD.html]

 


 **TOLC** and **HLYD** 
  
Found 21 abstracts in PubMed, retrieved 05.  
 

 What does Google say? 
 PDF only 
| .edu only 

---

**Interactive relationship** (e.g. stimulation, inhibition, etc)

**Non-interactive relationship** (e.g. studied together, co-existance, homology, etc.)

- The structure and function of the components of the HlyA secretion apparatus, HlyB,  **HlyD**  and  **TolC** , have been studied in great detail.  Ref: 11755084 Trends Microbiol, 2002
- In contrast, when the charged amino acid residues were deleted, the substrate was still engaged by  **HlyD**  but engagement was unproductive, i.e.  **TolC**  recruitment was not triggered.  Ref: 11676535 J Mol Biol, 2001
- Our results are compatible with the  **HlyD**  cytosolic domain mediating transduction of the substrate binding signal directly, presumably to the  **HlyD**  periplasmic domain, to trigger recruitment of  **TolC**  and assemble the type I export complex.  Ref: 11676535 J Mol Biol, 2001
- These data suggest that the HlyB  **HlyD**   **TolC**  based antigen delivery system with attenuated r S. typhimurium as carrier is capable of inducing an immune response against mycobacterial antigens.  Ref: 10727883 FEMS Immunol Med Microbiol, 2000
- This complex comprises a traffic ATPase HlyB and the 478 residue adaptor protein  **HlyD** , which contacts  **TolC**  during recruitment.  Ref: 11676535 J Mol Biol, 2001
- We show that an scFv HlyA hybrid secreted by Hly type I transporter  **TolC** , HlyB,  **HlyD**  is accumulated in the extracellular medium with the disulphide bonds correctly formed.  Ref: 11309117 Mol Microbiol, 2001
- In addition, we report on our r Salmonella typhimurium expression system combined with the HlyB  **HlyD**   **TolC**  export machinery for delivering the prominent mycobacterial antigen Ag85B for immune recognition.  Ref: 10076914 FEMS Immunol Med Microbiol, 1999
- A recombinant r Salmonella typhimurium aroA vaccine that secretes the naturally secreted protein of Mycobacterium bovis strain BCG, Ag85B, by means of the HlyB  **HlyD**   **TolC**  export machinery termed p30 in the following was constructed.  Ref: 10727883 FEMS Immunol Med Microbiol, 2000
